# Nature Studies—VII

**Published:** 1910-08-13

**Authors:** 


### The Practitioner's Relaxations.

### NATURE STUDIES—VII.

By a MAJOR R.A.M.C.

**The Way to Observe Wild Things—Second Broods—A Young Family—A Queer Pair of Friends.**

My daily task takes me on horseback across the highest near ridge of the downs. Every morning I use the same track, so I get to know the birds and beasts along my way and look out for them. Excepting by patient watching from hiding, there is no way of seeing wild things so good as riding by them on a quiet horse. The man on horseback is not seen so readily as the man a-foot; the presence of the horse allays suspicions.

Pheasants and partridges hatched well, but the spell of cold and wet just after Midsummer Day caused loss to the latter. But tragedies are common among wild things, and little regarded. If eggs or young are destroyed, a second brood is soon started. Tragedies, though, have been beyond the average near my house and along my track. One day two poaching dogs came along and played havoc. They startled several partridges from their nests and destroyed the eggs of some of them. They also slew a hare. Another day a prowling fox took six more.

The luck of some birds is wonderful. A hen pheasant chose for her nesting-place a bunch of nettles by the roadside about two hundred yards from my house. Here she laid and sat, not a yard from the high road and about a foot below it, for the nettles grew on the roadside bank of a ditch. I used to look down at her often as I passed by, and all day long dogs and various traffic used to go by her; yet there she sat undiscovered till the days were accomplished and she led off her brood to the standing corn. Keepers say a sitting bird has no scent. This is not quite true, for I have often seen a dog go up wind straight to a nest; but sitting, as birds do, quite still, crouched low among herbage, less scent is given off than if the bird were moving, and what there is probably rises on still days and is wafted only in a thin thread if there be wind. A factor that makes for the safety of sitting partridges is, I am certain, the constant near presence of the cock bird. The cock spends much of his time near by the nest, and the dog or fox that, getting wind of partridge, begins to hunt near the nest does not easily find the close-sitting hen, but springs her mate and is drawn off in vain pursuit. My paddock is a chosen haunt of partridges, but my terriers scarcely trouble now to hunt the line of them, frequent failures having disgusted them of the pursuit. Possibly foxes that have been frequently disappointed in a pounce at a cock bird may lose keenness and neglect the partridges for more easily caught young rabbits.

A partridge made a late nest in my paddock a little out from the hedge. She was sheltered by a clump of nettles, and I got the keeper to secure her still more by putting thorns on either side of her. She sat tight during the process of bushing her in. My dogs never found her, though they often moved her mate. I used often to visit her and to take children to see her, but she never got off her nest, though she sometimes hissed a protest. She had but few eggs under her, I think, for she left but few shells when she hatched. She drew off her brood through the hedge into high grass. I have not seen her since.

I have many times wondered what happens to a bird, such as the partridge, that lays a large clutch if her nest is destroyed when some only of her eggs are laid. One would suppose the need to lay pressing, yet, the nest gone, time is wanted to make another, even as scanty as a partridge's nest. I thought this nest in my paddock was perhaps made by one of the birds that was disturbed by the poaching dogs, for she came and made it very suddenly and unexpectedly, and had, I think, very few eggs, so perhaps only the remains of her clutch. I saw a pretty sight on July 14 as I rode home along the edge of newly seeded and rolled land. I suddenly became aware of a pair of partridges just under my left foot, and looking down I saw the heads of half a dozen little fellows watching me through their mother's feathers. The family had been dusting and basking; I had come unexpectedly upon them, so they had crouched, and as my horse kept steady pace past them they did not move at all.

Though the possession of a mate depends largely on fighting power, yet that is not all. Nearly every day I see a cock bird that is now the father of a fine covey though he has a very crippled leg. His mate sat near a gateway through which I turn to reach the down, and I used to see him often near by. So crippled is he that he can run but lamely and slowly, stumbling and fluttering as he goes, and as he flies his leg hangs. His misfortune must have been a heavy handicap in mating fights. That he won his mate and that she has remained faithful points to preference not based wholly on his prowess.

Till the clover and the corn grew high I used to see in the field before my house a strange pair of friends—a cock pheasant and a partridge that had chummed together. For two months, nearly every day, I used to watch these two feeding together, walking about the clover field, the partridge going in front, the pheasant closely following its every turn about the field, as closely as a dog after its master. Sometimes a pair of partridges that used the same haunt fed with these two, but it was easy to know the pheasant's friend from the other partridges by the closeness with which the pheasant followed it. As the clover grew I could see the pheasant walking in it, but often it would be a long while before I could get a glimpse of the smaller bird, though I could tell its position by the movements of its friend. Now the crops are high I have lost sight of them. The guess I make is that both had been wounded in the shooting season, and by reason of their wounds had been debarred from mating, so had chummed for mutual consolation. Both of them looked healthy, and the pheasant was in fine plumage. I think they roosted together in the open fields, for I used to see them early and late, and I never saw the pheasant go off to the cover.

